# Household expenditure on control of urban mosquitoes *Aedes albopictus* and *Culex pipiens* in Emilia-Romagna, Northern Italy

**DOI:** 10.1371/journal.pntd.0012552

**Published:** 2024-10-09

**Authors:** Massimo Canali, Laura Vici, Stefano Rivas Morales, Luciano Donati, Carmela Matrangolo, Claudio Venturelli, Paola Angelini, Michele Dottori, Romeo Bellini, Marco Carrieri

**Affiliations:** 1 Dipartimento di Scienze e Tecnologie Agroalimentari, Università di Bologna, Bologna, Italy; 2 Department of Statistical Sciences, University of Bologna, Bologna, Italy; 3 Centro Agricoltura Ambiente “G. Nicoli”, Crevalcore, Italy; 4 Local Public Health Unit, Public Health Department, Cesena (FC), Italy; 5 Settore Prevenzione collettiva e sanità pubblica Regione Emilia-Romagna, Bologna, Italy; 6 Istituto Zooprofilattico Sperimentale della Lombardia e dell’Emilia-Romagna “Bruno Ubertini”, Brescia, Italy; Brandeis University, UNITED STATES OF AMERICA

## Abstract

**Background:**

In 2007, the first outbreak of Chikungunya in Italy generated great alarm, highlighting the health risks caused by exotic species recently introduced in Europe and the need to strengthen control actions against the vectors. Besides health risks, mosquitoes cause nuisance, and citizens are required to adopt control measures. While the economic aspects of mosquito control by public agencies have been investigated, the scientific literature on the costs of mosquito protection incurred by families is scarce.

This study assessed the households’ expenditure on protective measures against mosquitoes in Emilia-Romagna, a region in Northern Italy.

**Methodology/Principal findings:**

A phone questionnaire survey was conducted to collect data on the annual expenditure incurred by households for self-protection against mosquitos in relation to the perceived level of nuisance and the household and dwelling characteristics. Univariate and multivariate analyses were conducted to identify the main determinants influencing such expenditure, which resulted affected by dwelling characteristics, presence of children under 6 years of age, and health concerns of family members. The average annual household expenditure was estimated at 84.63 euros, about 30 times higher than the expenditure per household supported by regional and local administrations for interventions against mosquitoes in public areas, as calculated in a previous study.

**Conclusion/Significance:**

Household expenditure is mainly aimed at providing a direct defense against mosquito bites (mosquito nets, adulticides, skin-on repellents, etc.) while spending for more effective measures addressed to reduce mosquito density results marginal: e.g., only 3.5% of the total expenditure was dedicated to larval control.

Control activities that lower the mosquito density in both private and public areas could reduce the use of household insecticides in urban environments and the related costs, and the risk of spread of imported arboviruses as well.

## Author summary

To achieve effectiveness, mosquito control requires the adoption of integrated pest management (IPM) methods and comprehensive strategies in both public and private areas.

In the case of urban mosquitoes, the application of IPM in public areas alone may not reduce the insect population density to levels that limit the nuisance, particularly during daytime, and mitigate the risks of arbovirus outbreaks.

In Italy, the control activities by local administrations are usually limited to public areas, while interventions in private areas are delegated to owners and residents. This study analyzed the expenses incurred by households to protect themselves from mosquitoes. We found that the average yearly cost per household was about € 84.63 (excluding the depreciation costs of mosquito nets, the average expenditure drops to € 58.63 per household), which was significantly higher than the average expenditure incurred by local administrations for their interventions in public areas (approximately € 3/household/year).

The efficacy of domestic mosquito control methods is difficult to estimate due to the variety of strategies, tools and operating conditions. Moreover, the application of domestic tools is mainly driven by marketing instead of sound cost-effectiveness evaluations.

## Introduction

In Italy, two harmful mosquito species are largely present in urban areas: the common house mosquito, *Culex pipiens* L., and the Asian tiger mosquito, *Aedes albopictus* (Skuse), which was introduced in Europe at the end of the 1970s [1]. Public health implications and nuisance caused by these mosquitoes require the implementation of specific prevention and control measures [2].

In Italian towns, before the introduction of *Ae*.*albopictus*, the most important mosquito species was *Cx*. *pipiens* and control activities carried out by public administrations were limited to sporadic adulticidal interventions in public areas. The principal control measures in private dwellings were the use of home insecticides and mosquito nets for windows. The establishment of *Ae*. *albopictus*, which is a highly anthropophilic, exophilic and diurnal species, has made all these measures inadequate and markedly modified citizens’ behavior in the use of courtyards and public and private gardens [3] [4].

In summer 2007, the first European epidemic of Chikungunya, an infectious disease caused by an arbovirus vectored by *Ae*. *albopictus*, affected several towns in Emilia-Romagna (ER), a region in Northern Italy [5]. This event caused great alarm all over Europe and raised public awareness of the associated health risks and the need to strengthen actions against the Asian tiger mosquito.

Furthermore, a constant and intensified circulation of the West Nile virus (WNV), an RNA virus vectored by *Cx*. *pipiens* that causes West Nile fever, was reported in Italy following the first human cases registered in 2008. In 2018, the peak of outbreaks was observed in Europe with about 2,000 cases, of which 576 in Italy [6].

In 2008, the public health services of the regional administration of Emilia-Romagna (RER) drafted the first “Regional Plan” for the fight against the Asian tiger mosquito and the prevention of chikungunya and dengue fevers. For its influence on the subsequent development of Italian policies for control and surveillance of arbovirus vectors, this Plan can be considered a precursor of the current National Arbovirus Surveillance and Control Plan (PNA) (www.salute.gov.it/imgs/C_17_pubblicazioni_2947_allegato.pdf). The first RER Plan devoted economic resources to specific studies and systematic quantitative monitoring of *Ae*. *albopictus* in the region and provided technical and financial support to municipalities and local health authorities (LHAs) in their efforts to control these mosquitoes. In 2012, the RER public health services also performed a cost analysis on the implementation of the Plan, finding that over the period 2008–2011, an average of €1.3 per inhabitant/year of public money from the budgets of municipalities and the RER was spent in the region for the tiger mosquito monitoring and control [7].

In Northern Italy, the current mosquito control activities, as conducted in public urban areas only, cannot adequately contain the nuisance caused by *Ae*. *albopictus* nor reduce the health risks [8]. Therefore, new tools are under development and evaluation [9].

Local administrations have issued major ordinances requiring citizens to control mosquito breeding sites in their private dwellings, but despite many years of implementation, the level of compliance remains very low and community participation is far from effective.

In the scientific literature, few studies examined private expenditure for mosquito control, and these studies only cover countries or regions where these insects may create serious health issues. According to the comparative evaluations of Legorreta-Soberanis et al. (2017), the household monthly expenditure on personal protection against mosquitoes, estimated as 2012 USD_PPP_ value (PPP = purchasing power parity), was 0.70–12.53 USD_PPP_ in Sri Lanka (2007), 1.1 USD_PPP_ in Tanzania (2009), 5.90–8.13 USD_PPP_ in India (2007), 10.43 USD_PPP_ in the Gambia, 12.13 USD_PPP_ in Mexico (2012), and 13.75–86.13 USD_PPP_ in Thailand (1999) [10]. In 2017 in Pakistan, the annual per capita private expenditure on mosquito prevention was between 47 USD and 94 USD [11].

On the island of La Reunion, the average monthly household expenditure was estimated at about 13.60 USD in 2012. This figure was influenced by subjective (e.g., respondents’ perception of the health threats from Chikungunya outbreaks, age, economic status and level of education) and objective factors (e.g., mosquito density) [12].

Our study aimed to help fill this knowledge gap by quantifying and analyzing the costs incurred by households for mosquito control in Emilia-Romagna. This region of Northern Italy can be considered representative of the ecological and residential context of the Po Valley, an area with high urbanization and a high level of economic development, whose environment and climate generally present suitable conditions for the development and spread of these insects. The information and data elaborated by our analysis were collected using a questionnaire administered to a representative sample of the regional population through phone interviews.

## Materials and methods

### The rationale behind the elaboration of the questionnaire utilized in the survey

For the elaboration of the questionnaire, we assumed that household expenditure for protection against tiger mosquitoes depends on three main drivers:

- the perception of the nuisance caused by mosquitos;
- the fear of possible diseases vectored by tiger mosquitoes;
- the nuisance caused by other insects (wasps and flies) or other types of mosquitoes for which people incur expenditure in common with those for tiger mosquitoes.

The perception of the nuisance caused by tiger mosquitoes was assumed to be related to objective and subjective variables. The objective variables refer to the characteristics of the household dwellings, such as the type of house (e.g., detached house, apartment in condominium building, etc.), the floor level of the apartment (e.g., ground, first, second, third floor, etc.), the presence of a courtyard and the level of its use by dwellers, the house location (e.g., city center, periphery, countryside), and the geographical position (in the case of the examined region, the altitude was deemed relevant). The subjective variables were related to certain characteristics of the household, in particular, the number of household members and the presence of children under 6 years of age.

**Fig 1:**
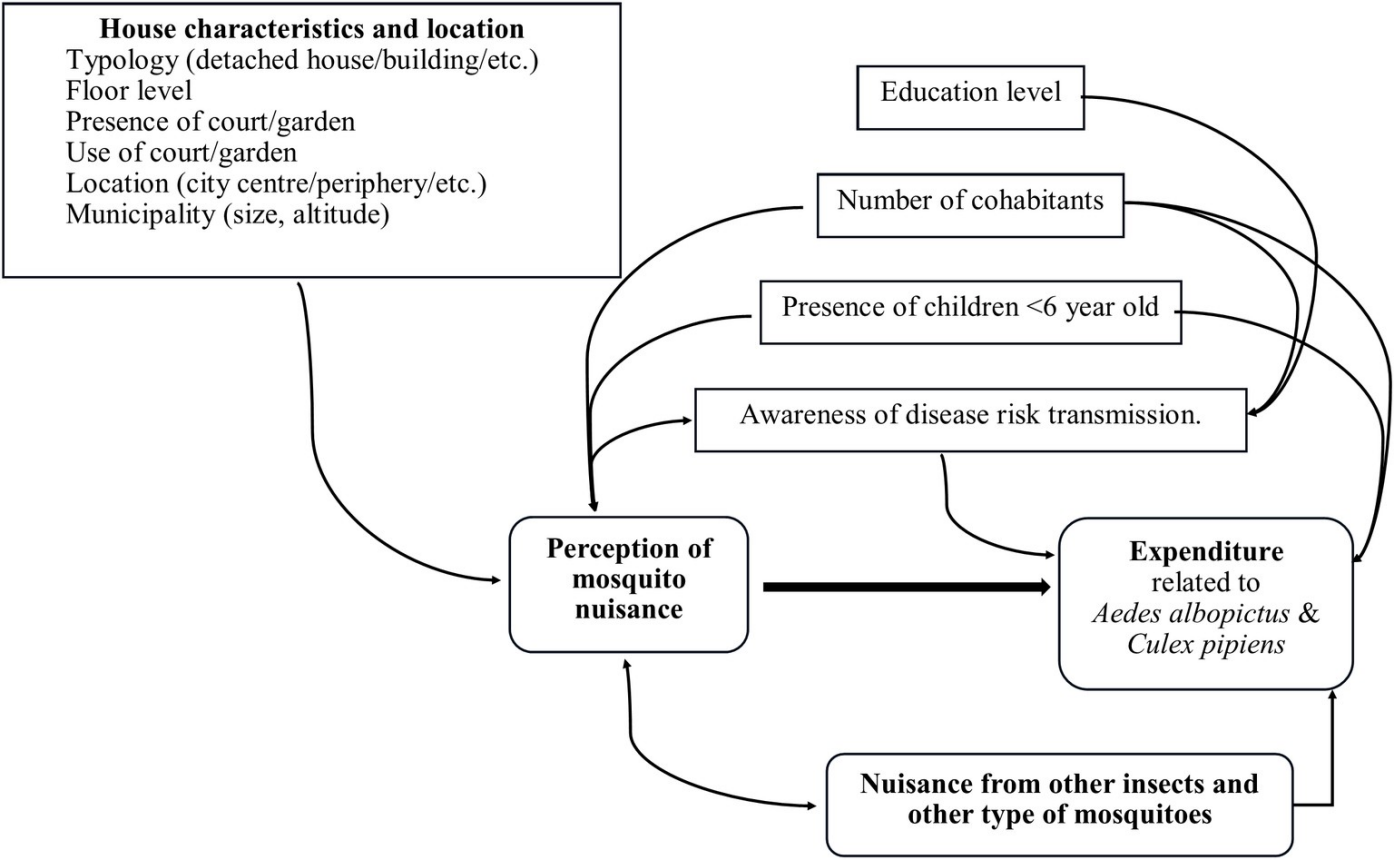
Objective and subjective variables considered for the elaboration of the questionnaire used in the survey.

Such subjective variables were also expected to directly influence the household’s expenditure and, together with the education level of household members, the concern for the health risks associated with tiger mosquitoes. Fig 1 summarizes the variables and the respective interrelations considered for the elaboration of the questionnaire utilized in the survey.

On this basis, the questionnaire was formulated to collect information on the following elements:

- the main environmental and social variables that influence the people’s behavior regarding tiger mosquito control (e.g., type and location of the house, level of education, number of household members, presence of children under 6 years of age, etc.);
- the level of nuisance from mosquitoes as perceived by household members;
- the level of concern regarding the associated health risks;
- the measures implemented by households to protect themselves from mosquitoes and the suitability and efficacy of the measures;
- the expenditure incurred by households on such measures.

The questionnaire was designed to be administered through telephone interviews. An English translation of the questionnaire is provided in S1 Table.

### Sampling of the interviewees

According to the logistic and financial conditions for the survey, the sample for the phone interview was randomly extracted from the population registries of RER Local Health Authorities (LHAs).

The extraction followed three stratifications:

- territory of LHAs (situation before the year 2014)
- demographic size of municipalities (data on Jan. 1^st^, 2014):
  ○ Municipalities with < 10,000 inhabitants;
  ○ Municipalities with 10,000–50,000 inhabitants;
  ○ Municipalities with > 50,000 inhabitants;
- age of respondents (people born before 1^st^ Jan. 1995).

The extracted sample included 1,391 individuals from the ER adult population born before Jan. 1^st^, 1995, distributed according to the population of residents in the territory of the selected LHAs and in the three size categories of municipalities (as shown in the S2 Table). The provinces of Piacenza, Modena and Ferrara were not included in the survey because of logistical reasons.

### Interviews

A total of 412 interviews were granted by the 1391 individuals sampled.

The interviews were conducted by phone, following a communication letter sent by the LHAs to all sampled residents. The interviewees were called through their home phones or cell phones, depending on the type of contacts they had provided to the LHAs’ registries. We have made intensive efforts to enlist the cooperation of respondents with a low response propensity and, achieve a response for all sampled units minimizing nonresponse bias (e.g., unresponsive telephone numbers were called back up to four times before giving up on the interview, those who did not have time for the interview at the time of the call were called back at times convenient to them, those who were unable to answer about the expenses incurred for mosquito repellent products were asked to have the interview carried out with the person in the household who was responsible for these purchases; etc.).

The interviews progressed in parallel in the different categories of municipalities, as follows:

- 57 interviews were performed in 2015 to collect information related to the 2014 season;
- 355 interviews were performed in 2017 to collect information related to the 2016 season.

### Costs

The average annual expenditure on mosquito control per household was calculated. When the respondents were not able to specify the expenditure for a given item, this was estimated by applying the following criteria:

- If the respondents declared the type and quantity of the products utilized, this was multiplied by the average price of the product obtained from the survey. For those who used a given type of product without indicating the quantity used, the median of the expenditure for this product recorded in the survey was considered.

- For the cost of mosquito nets, which are durable goods, an amortization period of 20 years was assumed;

- For the expenses incurred in the context of common properties, e.g., disinfestation in condominium areas, we considered the average costs resulting from the survey.

S1– Data reports the dataset obtained from the interviews.

### Statistical analysis

Univariate, bivariate and multivariate analyses were performed to explore the context and behaviors of households to control mosquitoes. Angular transformation of the percentages and log transformation of the costs were used. Linear regressions with robust standard errors were estimated to identify the variables influencing expenditure on household self-protection against mosquitoes. Tukey’s test was used to find the averages that were significantly different from each other. The paired two-sample t-test allowed comparisons between diurnal and nocturnal nuisance.

## Results

A total of 412 interviews were done, corresponding to a response rate of 29.6% of the initial sample.

The territorial distribution of the interviews is shown in Annex 3. Despite the relatively low rate of respondents, the geographical distribution of the interviews was not significantly different from the distribution of the total population in the territories of the analyzed LHAs. Major differences were an over-representation by 1.2% of the interviews made in the municipalities of the Reggio Emilia LHA with population between 10,000 and 50,000 inhabitants, and an under-representation by 1.1% of the interviews made in the municipalities of the Bologna LHA with population of more than 50,000 inhabitants. Regarding the territories under the jurisdiction of the LHAs, the LHA of Imola was the most over-represented in the distribution of the interviews with +1.1% compared to its total population, and Parma the most under-represented with -2.0%. Considering the stratification by group of municipalities, those with population between 10,000 and 50,000 population were over-represented by 0.8%, and those with more than 50,000 population were under-represented by 1.0%.

### Respondents’ characteristics

The average age of the respondents was 55.75 years (SD 16.88), the minimum age was 19, and the maximum was 90. About 43.39% of the interviewees were male and 54.61% were female.

Regarding the educational background of the interviewees, approximately 11% completed primary school, nearly 26% completed secondary school, around 42% completed high school, approximately 3% had a Bachelor’s degree, and about 17% had a master’s degree or higher. Only 0.5% declined to divulge their highest level of education.

The survey findings indicate that 84% of the interviewed households had no children under 6 years of age with a median size of two members. On the other hand, households with children under six years accounted for 16% of the total and had an average size of four members.

In terms of housing, approximately 45% of the interviewees lived in apartment buildings, while nearly 21% lived in multi-family apartments. Approximately 20% lived in independent houses, and about 14% lived in other house types.

Regarding the location of their residence, 45% of the respondents lived in the suburbs around the city centers, almost 30% lived in city centers, about 20% lived in the countryside near urban areas, and less than 5% lived in other locations. About 85% of respondents had gardens: of this figure, 43% used them frequently, 27% only on weekends, and the remaining 30% rarely.

### Expenditure according to the size of municipalities and dwellings

There were no significant differences in the total annual expenditure per household (including mosquito nets) according to the size of the municipalities of the interviewees (F_2,409_ = 0.65 and p = 0.52), even if a greater expenditure was observed in smaller cities (see Table 1).

**Table 1:** Annual expenditure per household according to the size of the municipalities of the interviewees.

Population size	N	Mean (€)	Median (€)	SD (€)	Percentile (€)		
					25^th^	50^th^	75^th^
<10,000	93	91.49	68.70	85.83	40.00	68.70	114.50
10,000–50,000	136	87.05	62.00	102.83	37.50	62.00	103.31
>50,000	183	79.35	57.18	77.94	25.27	57.18	115.13
Total	412	84.63	61.50	88.42	32.00	61.50	112.50

The average annual expenditure incurred by the interviewees was €84.63 per household, with a median value of €61.50 per household. The 25th percentile of the expenditure value was €32.00 per household while the 75^th^ percentile was €112.50 per household.

Excluding the amortizationcosts of the mosquito nets, the average expenditure drops to €58.63 per household, with a median value of €33.00 per household. The 25th percentile expenditure was €12.50 per household while the 75th percentile was €75.00 per household.

### Products purchased by households

The subdivision of costs into the various product categories purchased by families is shown in Table 2.

**Table 2:** Annual expenditure per household for different anti-mosquito products.

Type of product	No. respondents	No. positive answers	Mean (€)	SD (€)	% of total expenditure
Insecticide spray products for indoor or outdoor usage	412	132	5.54	13.38	6.55
Repellent tablets and other electric diffusers	412	156	8.41	26.04	9.94
Repellents for outdoor use (i.e., mosquito coils or vaporizers)	412	166	6.98	13.05	8.25
Natural repellents for on-skin use	411	128	6.12	13.42	7.23
Chemical repellents for on-skin use	412	172	6.31	11.40	7.46
Electric, CO_2_, pheromones, triggering or ultrasonic traps	411	35	3.96	17.70	4.68
Larvicidal products for water containers	403	174	2.93	7.94	3.46
Adulticides	412	38	9.49	12.73	11.21
Other products against mosquito bites	412	54	1.80	2.26	2.13
Mosquito nets (20-year amortization)	412	255	25.91	30.75	30.62
Condominium interventions (larvicide and adulticide treatments)	412	96	7.03	25.64	8.31
Total	412	392	84.48	88.42	6.55

### Expenditure in condominium buildings

Only 194 of the 412 interviewees (47.09%) living in condominium buildings (ed.: with more than 4 apartments) and 96 (49.48%) spent money to protect their households against mosquitoes. About 93.75% of the 96 carried out larvicidal interventions in the drains and 34.38% used adulticide products in courtyards and gardens. Regarding adulticide treatments, 9.38% practiced spot interventions, while 25.00% carried out calendar interventions. The average expenditure incurred by the 96 interviewees who supported some spending was €30.16 (SD €46.24) while the average of expenditure in condominium buildings was €7.03 (SD €25.64)

### Households practicing adulticide treatments in private courtyards and gardens

In total, 38 interviewees (9.22%) performed adulticide treatments, of which 17.86% lived in single-family detached houses, 17.44% lived in apartments in multi-unit dwellings, 15.30% lived in condominium buildings, and 22.03% lived in other types of dwellings. No statistically significant differences were found in adulticide treatments across different housing typologies (Table 3).

**Table 3:** Adulticide treatments in private gardens according to the different types of dwellings.

Type of dwelling	Respondents (No.)	Practicing adulticide treatments (No.)	% respondents
Detached house	84	12	14.29
Apartment in multi-unit dwelling	86	9	10.47
Apartment in condominium building	183	8	4.37
Other	59	9	15.25
Total	412	38	9.22

### Expenditure by type of dwelling

The households living in detached houses and apartments in multi-unit dwellings declared significantly higher expenditure than those living in condominium buildings (F_3,408_ = 9.20 and p<0.001), by spending more on insecticide spray products for indoor or outdoor treatments (F_3,408_ = 5.79 and p<0.001), mosquito traps (F_3,407_ = 4.76 and p<0.003), larvicidal products (F_3,399_ = 7.85 and p<0.001), and mosquito nets (F_3,408_ = 9.89 and p<0.001) (see Table 4).

**Table 4:** Expenditure per household according to the dwellings of the respondents.

Type of dwelling	No	Mean (€)	Median (€)	SD (€)	Percentile (€)			Tukey’s Test
					25th	50th	75th	
Detached house	84	102.11	63.42	105.28	37.5	63.42	128.11	a
Apartment in multi-unit dwelling	86	116.91	97.75	108.55	61.75	97.75	145.62	a
Apartment in condominium building	183	62.69	48.5	63.91	20	48.5	75.89	b
Other	59	80.77	64.18	77.93	37.5	64.18	104.49	b

### Expenditure by households having private courtyards and gardens

**Fig 2:**
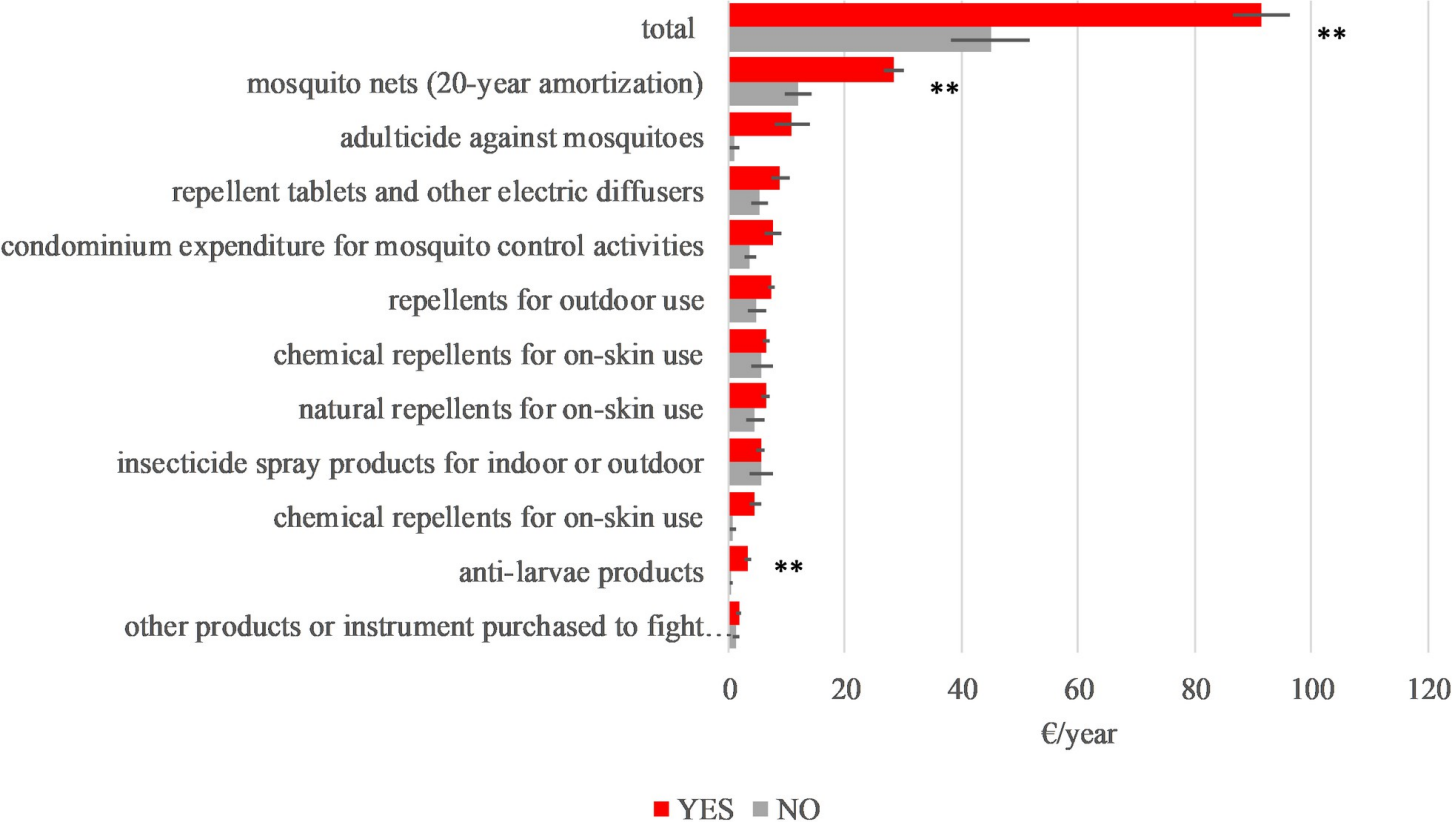
Expenditure of respondents according to presence (YES) or absence (NO) of private gardens in their houses (Tukey Test ** p<0.01 *p<0.05).

The households having private courtyards or gardens spent significantly more (91.38 ± 91.66 €) for mosquito control (F_1,410_ = 14.48, p<0.001) than those who have not. Most relevant spending differences were found for larvicidal products (3.35±8.46 €) and mosquito nets (565.68±38.90 €) (F_1,401_ = 6.64, p<0.01; and F_1,410_ = 14.86, p<0.001 respectively) (Fig 2).

### Presence of children under 6 years of age in the household

**Fig 3:**
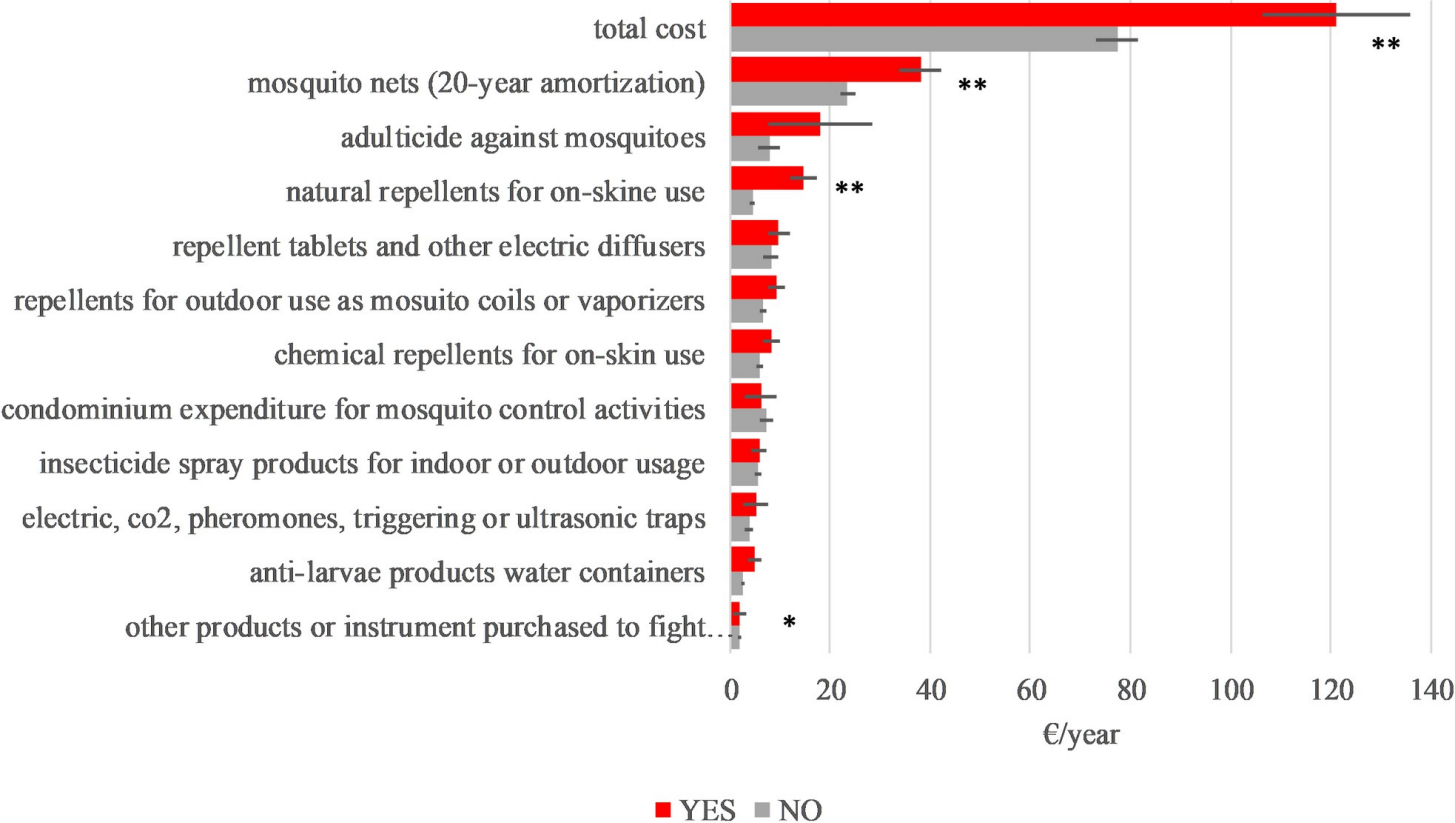
Respondents’ expenditure according to the presence (YES) or absence (NO) of children under 6 years of age in the households (Tukey Test ** p<0.01 *p<0.05).

In the households with children under 6 years of age, a significantly higher level of expenditure (about 1.6 times more than the average) was incurred (F_1,410_ = 14.39 and p<0.001). The higher expenses were due to a greater use of natural repellents (F_1,409_ = 34.48 and p<0.001), larvicidal products (F_1,401_ = 4.97 and p<0.03) and mosquito nets (F_1,410_ = 13.04 and p<0.001) (Fig 3).

### Perceived nuisance from insects

**Fig 4:**
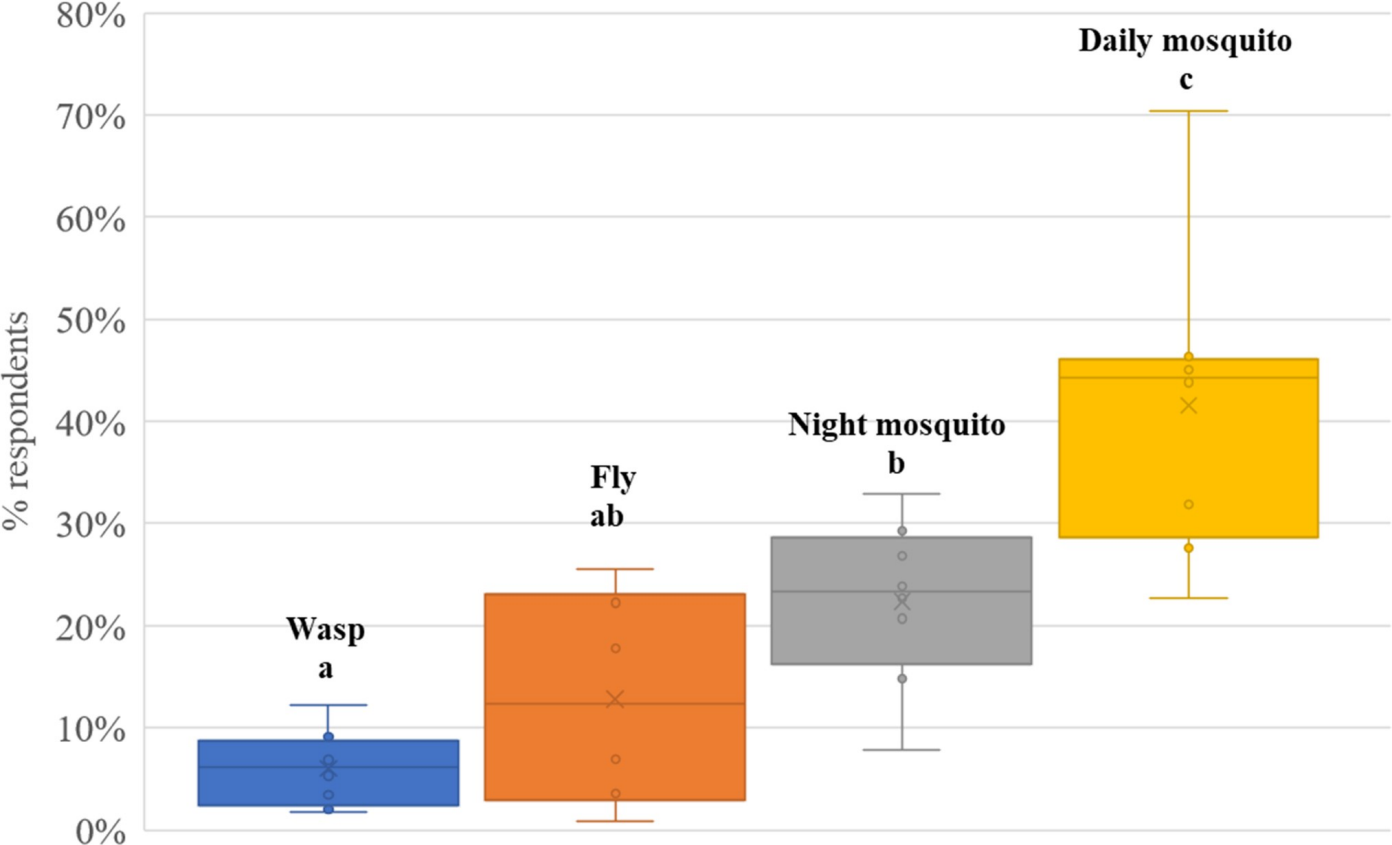
Average levels of nuisance caused by the examined insects in the territory of the LHAs included in the survey.

Regarding the level of perceived insect nuisance (including flies, wasps, and night- and daytime-biting mosquitoes), significant variations were found in the territory of the LHAs included in the survey (F_3,26_ = 18.63 and p<0.001) (Fig 4).

Wasps and Flies. There were low complaints by respondents regarding nuisance caused by wasps and flies. We observed significant differences in the level of the nuisance caused by flies (F_7,404_ = 8.03 and p<0.001) between LHAs, with greater nuisance levels recorded in the areas with a higher density of animal farms (Parma, Reggio Emilia, Cesena and Forlì), where 18% to 25% of respondents experienced a substantial nuisance.

Mosquitoes. Significant differences were observed between the levels of the diurnal and nocturnal nuisance from mosquitoes experienced by respondents (t = -7.56 DF 410 p<0.001).

About 22.36 ± 8.03% of the respondents attested to suffer mosquito nuisance at night (likely caused by *Cx*. *pipiens*), while 41.68 ± 14.36% during the day (likely from *Ae*. *albopictus*) (Fig 4). Almost 42% of respondents were very annoyed by the presence of both diurnal and nocturnal biting mosquitoes, and, consequently, significantly higher expenditure was incurred to control them (diurnal bites F_2,408_ = 36.99 and p<0.001; nocturnal bites F_2,409_ = 6.31 and p<0.002) (Table 5).

**Table 5:** Relationship between the perceived nuisance from diurnal and nocturnal biting mosquitoes and the expenditure in euros per household.

Nuisance level	Diurnal bites (*Ae*. *albopictus*)			Nocturnal bites (*Cx*. *pipiens*)		
	No	Mean expenditure ±SD (€)	Tukey test	No	Mean expenditure ±SD (€)	Tukey’s test
Low	108	44.30±48.63	a	150	69.66±93.51	a
Medium	136	67.17±56.54	a	165	83.32±71.76	a
High	167	125.33±110.82	b	97	110.03±100.90	b

Nuisance caused by nocturnal mosquitoes was not related to the presence of private gardens or courtyards, or children under 6 years of age in the house, while nuisance by diurnal mosquitoes was significantly correlated to such factors (F_1,409_ = 13.13 and p<0.001 for the presence of private gardens or courtyards and F_1,409_ = 12.57 and p<0.001 for presence of children under 6 years of age).

### Concern about the vectorial capacity of tiger mosquitoes

Only 14.56% of respondents considered Tiger Mosquito a potential health risk. However, the expenditure incurred by households on mosquito control was highly correlated with the concern about the vectorial capacity of *Ae*. *albopictus* (F_2,408_ = 18.08 and p<0.001). This expenditure ranged from €60.51 ± 58.12 per household when the concern was low to €134.92 ± 119.82 per household when it was high (Table 6).

**Table 6:** Correlation between the concern about the vectorial capacity of tiger mosquitoes and the expenditure per household.

Level of concern	No.	Mean expenditure (€/household)	SD (€)	Percentile (€/household)			Tukey’s test
				25^th^	50^th^	75th	
Low	176	60.51	58.12	20.00	46.56	75.00	a
Medium	175	91.33	93.40	41.25	72.00	118.53	b
High	60	134.92	119.82	53.40	89.67	179.86	c

### Multivariate analysis

The results of the multivariate analysis on the total expenditure incurred by the interviewed households are summarized in Table 7. In Column 1 of this table, the full sample is analyzed. The independent variables that positively and significantly affected the total expenditure were the presence of a private courtyard or garden, the number of household members, the perceived nuisance from mosquito bites, and the level of health concern.

**Table 7:** Linear regressions (OLS with robust s.e.) of total mosquito control costs (in log): full sample, with mosquito nets, and without mosquito nets.

	(1)	(2)	(3)
Independent variables	Full sample	With mosquito nets	Without mosquito nets
Floor number	-0.044	0.008	-0.078
	[0.036]	[0.049]	[0.052]
External garden YN	0.624***	0.445**	0.925***
	[0.173]	[0.175]	[0.254]
Number of inhabitants	0.106**	0.001	0.300***
	[0.048]	[0.043]	[0.099]
Presence of children	0.049	0.043	0.389
	[0.150]	[0.167]	[0.290]
Urban centre	-0.003	0.732	-0.452
	[0.473]	[0.471]	[0.525]
Suburbs	-0.335	0.456	-0.864
	[0.474]	[0.473]	[0.544]
Countryside area	-0.147	0.543	0.143
	[0.474]	[0.477]	[0.577]
Other locations (ref)			
Nuisance lev. (ord)	0.437***	0.313***	0.459***
	[0.077]	[0.079]	[0.161]
Health concern (ord)	0.216***	0.146*	0.287*
	[0.078]	[0.080]	[0.169]
Primary school (ref)			
Lower secondary school	0.049	0.028	0.226
	[0.193]	[0.161]	[0.386]
High school	0.011	0.352**	-0.430
	[0.188]	[0.154]	[0.358]
Bachelor degree	0.042	0.739***	-0.951
	[0.411]	[0.201]	[0.611]
Master degree	-0.016	0.277	-0.461
	[0.221]	[0.174]	[0.419]
Mosquito Nets YN	0.764***		
	[0.120]		
Constant	2.209***	2.647***	2.203***
	[0.518]	[0.510]	[0.656]
Observations	294	170	124
R^2^	0.426	0.284	0.408
Adj. R^2^	0.398	0.224	0.338
BIC	881.024	407.382	441.625

Standard errors in brackets

* *p* < 0.10

** *p* < 0.05

*** *p* < 0.01

Since the spending on mosquito control may also depend on the presence of mosquito nets, the sample was split to compare the households who had installed widow nets with those who had not. In the first case (Table 7, Column 2), the household expenditure resulted positively and significantly influenced by the presence of a courtyard or a garden, the perceived nuisance, and the level of health concern. Additionally, for these households, higher levels of education were also significantly associated with increased spending. Regarding the households who had not installed mosquito nets (Table 7, Column 3), the significant factors for household spending were the presence of a private courtyard or garden, the level of perceived nuisance and health concern (p < 0.1) and the number of household members.

Based on the baseline regression model, the predicted average household expenditure on mosquito control resulted €62.03, including also the households with no expenditure, and €64.36 for the households that supported some expenditure.

Additional tables for a more detailed analysis are available in S3–S5 Tables. These include: S3 Table, examining the impact of nuisance from different types of insects such as wasps, flies, tiger mosquitoes, domestic mosquitoes, and other insects on household expenditure; S4 Table, analyzing the factors impacting onhousehold expenditure without depreciation of mosquito nets; and S5 Table presenting a logit model that explores the factors influencing decisions on investments for mosquito nets.

Table 8 summarizes the results of a multivariate analysis of the expenditure for natural and chemical products incurred by the interviewed families. Several key differences can be observed when comparing the expenditure for the two product typologies. The presence of children under 6 years of age significantly influenced the purchase of natural products across all samples (with the highest impact observed in households without mosquito nets). The significant factors that increase spending on chemical products include the presence of a private courtyard or garden, the number of household members (in houses without mosquito nets), and the level of health concern. Perceived bite nuisance has a significant and positive impact on total spending on both chemical and natural products.

**Table 8:** Linear regressions analysis of expenditure (in log) on natural and chemical products (OLS with robust standard errors).

	Expenditure in natural products			Expenditure in chemical products		
	(1)	(2)	(3)	(4)	(5)	(6)
Independent variables	Full sample	With mosquito nets	Without mosquito nets	Full sample	With mosquito nets	Without mosquito nets
Floor number	-0.020	-0.006	-0.049	-0.028	0.020	-0.069
	[0.046]	[0.107]	[0.049]	[0.044]	[0.112]	[0.054]
External garden YN	0.166	-0.012	0.382*	0.678***	0.716*	0.843***
	[0.174]	[0.292]	[0.204]	[0.236]	[0.401]	[0.259]
Number of inhabitants	0.071	0.010	0.218**	0.101	0.011	0.286***
	[0.064]	[0.084]	[0.107]	[0.070]	[0.092]	[0.106]
Presence of children	0.889***	0.803***	1.373***	-0.049	-0.134	0.232
	[0.245]	[0.292]	[0.407]	[0.251]	[0.307]	[0.326]
Urban centre	-0.411	-0.897	-0.418	-0.233	0.268	-0.452
	[0.449]	[0.779]	[0.553]	[0.441]	[0.389]	[0.529]
Suburbs	-0.313	-0.939	-0.117	-0.573	0.072	-0.989*
	[0.454]	[0.768]	[0.574]	[0.442]	[0.340]	[0.548]
Countryside area	-0.322	-0.891	0.251	-0.316	0.206	0.070
	[0.487]	[0.789]	[0.732]	[0.466]	[0.328]	[0.583]
Other locations (ref)						
Nuisance lev. (ord)	0.562***	0.640***	0.412**	0.426***	0.394**	0.389**
	[0.101]	[0.136]	[0.159]	[0.115]	[0.169]	[0.167]
Concern lev. (ord)	0.038	0.027	0.057	0.337**	0.380*	0.268
	[0.121]	[0.160]	[0.185]	[0.136]	[0.193]	[0.167]
Primary school (ref)						
Lower secondary school	0.164	0.330	-0.075	0.123	0.262	0.092
	[0.267]	[0.353]	[0.405]	[0.311]	[0.462]	[0.415]
High school	0.353	0.665*	-0.076	0.236	0.825*	-0.563
	[0.257]	[0.345]	[0.373]	[0.291]	[0.440]	[0.372]
Bachelor degree	0.990**	1.903**	0.020	0.461	1.856***	-0.993*
	[0.493]	[0.794]	[0.526]	[0.495]	[0.457]	[0.587]
Master degree	0.391	0.669*	0.002	0.042	0.397	-0.563
	[0.288]	[0.394]	[0.421]	[0.326]	[0.497]	[0.421]
Mosquito Nets YN	-0.095			-0.346*		
	[0.169]			[0.180]		
Constant	0.151	0.622	0.000	1.994***	0.902	2.355***
	[0.512]	[0.892]	[0.661]	[0.533]	[0.669]	[0.652]
Observations	294	170	124	294	170	124
R^2^	0.245	0.238	0.320	0.160	0.156	0.329
Adj. R^2^	0.208	0.174	0.240	0.118	0.085	0.249
BIC	1049.617	643.469	443.496	1118.971	681.625	463.319

Standard errors in brackets

* *p* < 0.10

** *p* < 0.05

*** *p* < 0.01

## Discussion

### General considerations and comparisons with other studies

In our interviewed sample, the annual private expenditure for mosquito control ranged from €44 to €138 per household and was correlated to the characteristics of the dwellings, the presence of children under six years of age in the households, and the level of health concerns for mosquitoes’ vector capacity. Considering the existence of 2,029,000 households in ER [13] in 2014, the total regional expenditure of households was estimated at €171,673,690, corresponding to 0.1% of the regional GDP. The expenditure incurred by public administrations, i.e., the ERR administration, municipalities, and the LHAs in the region for the implementation of the Regional Control Plan was estimated, by a previous study, around €5.3 million or €1.3 per inhabitant, which is about 32 times lower than the household expenditure per inhabitant.

In Italy, the public health risks from mosquitoes relate to *Cx*. *Pipiens*, as a vector of the West Nile virus (WNV) (an endemic Flavivirus), and *Ae*. *albopictus*, as a vector of the Chikungunya virus (CHIKV), the dengue virus (DENV), and the Zika virus (ZIKAV, non-endemic in Italy). In our survey, the percentage of respondents expressing concern about the vector capacities of mosquitoes was relatively low (14.56% of respondents), while the percentage perceiving annoyance caused by these insects was significantly higher, especially regarding *Ae*. *albopictus*. However, the average monthly expenditure of families (considering five months from May to September) in ER resulted high (€16.92 or USD 18.27) and not dissimilar from the monthly expenses estimated in other countries where the circulation of arboviruses is more intense. For example, on the island of La Reunion, the monthly expenditure was estimated at 13.60 USD per household [12].

We found that much of the household expenditure was for measures protecting against mosquito bites. About 60% of respondents installed mosquito nets on windows, which defend against endophilic mosquitoes (mainly *Cx*. *pipiens*), and 62% bought on-skin repellents (of which 19.90% declared to buy only “natural” products, 30.58% only chemical, and 11.17% both types) that are generally against exophilic mosquitoes (especially*Ae*. *albopicuts*) in the analyzed region. While education was not always a significant predictor of mosquito control spending, our results indicate that higher education levels were associated with increased expenditure on mosquito control measures in households with mosquito nets. This finding suggests that although mosquitoes pose a universal risk across different economic and educational groups, better-educated households may be more proactive in investing in protective measures against this threat.

### Limitations and directions for future research

Our study cannot avoid the limitations of all the investigations that, for any reason, have to collect detailed economic data from consumers through call surveys. The information gathered relies on respondents’ statements, subjective evaluations, recollections of expenses incurred months or years before (as for mosquito window nets setting), and their technical knowledge of available mosquito control practices. Against these inconveniences, we sampled only adult respondents, ensured that the interviewees were the persons in households shopping for products and tools for mosquito control, cross-checked the technical data regarding the measures put in place by surveyed households with the declared expenditure and adjusted the values, by using the average of the values indicated by other respondents, when the economic data provided resulted unreliable.

We had to use a cross-sectional study design that restricts the ability to assess changes over time. Therefore, we could not analyze the monthly variations of household expenditure for mosquito control. Future studies will emphasize such variations more accurately.

Non-response bias is an error that occurs when a part of the sample does not participate in a survey and can result in the under-representation of some population groups, leading to biased results. In a study such as ours, the non-response could be linked to a lack of interest as the mosquito problem is not perceived by the interviewed, and this could lead to an underestimation of the cost.

The non-response in this type of surveys is unavoidable. In similar studies, the response rate was from 20% to 38% [14, 15, 16]. The adopted measures to minimize non-response bias included a presentation letter of the survey sent by the LHAs two weeks before the interview to the sampled households, reassuring respondents that the study would keep collected information completely confidential and trying to shorten the duration of the phone interviews as much as possible. Furthermore, at the first phone contact, the interviewer was available to call back at the time indicated by the interviewee to stimulate the cooperation of respondents with a low response propensity.

## Conclusions

Despite the recurrent information campaigns launched by the RER to promote the use of larvicide products to treat potential *Ae*. *albopictus* breeding sites in private gardens and courtyards, only 43.18% of respondents attested to purchasing such products, with an average yearly expenditure of €2.9 per household. Most expenses for mosquito control, i.e., 58.79%, were for chemical repellents and pesticides. Only 10.60% were for products with natural active substances, while 30.61% were for mosquito nets.

ER households spend a considerable amount of money on protection against mosquitoes, especially compared to the expenditure incurred by public administrations. However, the effectiveness of tools deployed by families appears to be limited. 78.35% of the interviewees reported experiencing medium to high annoyance from mosquitos (both diurnal and nocturnal). Additionally, 66.25% of respondents indicated that mosquitoes disrupted their garden enjoyment.

As a matter of evidence, in Italy, the mosquito control and prevention activities intended in the PNA and the protection measures undertaken privately by citizens are not sufficient to prevent the risks of disease outbreaks from imported viruses [17,18,19] and to contain the spread of the WNV [20]. The density of *Ae*.*albopictus* in ER, despite the expenditure incurred to citizens, is very high, and the bites per capita vary from 0.8 in spring to 5.9 during the summer (on average 3.73 bites per capita) [21].

The information collected by the survey may guide communication strategies aimed at improving the understanding of mosquito-related issues and promoting larval reduction activities in private areas while reducing the use of adulticides in the urban environment. Implementing strategically targeted information campaigns on good practices against urban mosquitoes may further decrease the risk of the accidental spread of imported viruses and the improper use of adulticide products which can favor resistance phenomena [22,23].

## Supporting information

S1 Data DB of data phone questionnaire. (XLSX)

S1 Table The phone questionnaire. (DOCX)

S2 Table Stratified samples of candidate citizen interviewees by Local Health Authorities (LHAs) territory and municipality size and Distribution of interviews by LHAs territory and municipality size. (DOCX)

S3 Table Linear regressions analysis of insect control expenditure (in log). The table shows the results of the linear regression analysis of total spending for insect control, considering factors such as the level of annoyance related to different types of insects such as wasps, flies, common and tiger mosquitoes. Apart from columns (1) and (2) which analyze the full sample, the estimates in columns (3) and (4) pertain specifically to the subsamples of households with and without mosquito nets at home, respectively. In the full sample analysis in column (1), which excludes concern and bites annoyance from tiger mosquitoes, significant factors influencing total expenditure include the presence of an external garden, and nuisance levels from flies and common mosquitoes. In the full sample analysis in column (2), which includes all insects, significant factors positively affecting total expenditure are the presence of an external garden and the level of nuisance from flies. Additionally, households that experience higher levels of nuisance and concern from tiger mosquitoes tend to spend more on insect control measures. For households with mosquito nets (column 3), significant factors are the presence of an external garden, the area where the dwelling is located, the presence of flies, and the nuisance level from tiger mosquitoes. Higher education levels also significantly increase spending. In analysing the expenditure incurred by households without mosquito nets (column 4), significant factors include the presence of an external garden, the number of inhabitants, and the nuisance level from tiger mosquitoes.
(DOCX)

S4 Table Linear regression analysis of total expenditure on mosquito control (in log) net of mosquito net costs (OLS with robust s.e.). The table presents the results of the linear regression analysis of total expenditure on insect control, excluding costs related to the installation of mosquito nets. In the analysis on the full sample (column 1), significant factors influencing total expenditure include the presence of an external garden, the number of inhabitants, the nuisance level from mosquitoes, and the level of concern about their bites. For households with mosquito nets (column 2), significant factors are the nuisance level from insects and educational attainment. In households without mosquito nets (column 3), significant factors are the presence of an external garden, the number of inhabitants, the nuisance level from tiger mosquitoes, and the level of concern about their bites. These results indicate that the presence of an external garden and nuisance levels from insects consistently and positively impact insect control expenditure across all samples, with higher levels of concern also playing a significant role, particularly in households without mosquito nets.
(DOCX)

S5 Table Logit model analysis of factors influencing investment in mosquito nets (binary dep. var.) The table aims to identify those factors affecting the decision to install mosquito nets and incur the associated costs. The estimation of the logit model reveals that the floor level negatively impacts this decision, suggesting that higher floors reduce the probability of purchasing nets. Conversely, living in a countryside area significantly increases the likelihood of investing in mosquito nets. Moreover, higher levels of nuisance from tiger mosquitoes and other insects positively influence the decision to install mosquito nets. Other factors, such as the presence of an external garden, the number of inhabitants, the presence of children, and concern level about tiger mosquitoes, do not appear to significantly affect the investment decision.
(DOCX)
