# Supplementary material for: Household expenditure on control of urban mosquitoes Aedes albopictus and Culex pipiens in Emilia-Romagna, Northern Italy
Source: PLoS Negl Trop Dis. 2024 Oct 9;18(10):e0012552. doi: 10.1371/journal.pntd.0012552 (PMC11537423; doi:10.1371/journal.pntd.0012552)
Supplement: S1 Table — (DOCX) [file pntd.0012552.s002.docx]

**S1 Table. The phone questionnaire**

| The questionnaire was organized to collect the following information: |
| --- |
| RESPONDENT |
| Sex |
| Age |
| Educational level |
| Main buyer of mosquito repellent products |
| FAMILIES |
| Number of cohabitants |
| Presence of children (<6 years) |
| FEATURES OF THE HOUSE |
| Typology (detached house / building / etc .) |
| Ground floor |
| Presence of court / garden |
| Use of the courtyard / garden |
| Location (city center / suburbs / etc .) |
| Altitude |
| INSECTS OTHER THAN ATM |
| Nuisance level of houseflies |
| Expenditure on houseflies (Y / N) |
| Nuisance of the wasp |
| Expenditure on wasps (Y / N) |
| Nuisance from other insects |
| Expenditure on other insects (Y / N) |
| The ordinary nuisance of mosquitoes |
| Expenditure on mosquitoes other than ATM (Y / N) |
| PERCEPTION OF ATM |
| **Nuisance level** |
| Expenditure on ATM (Y / N) |
| Discomfort when using gardens / parks / etc . |
| Concern about the risk for disease transmission |
| EXPENDITURE ON ATM |
| Insecticide spray products (no. / €) |
| Insecticide/repellents indoor electric devices (no. / €) |
| Coils and other outdoor products (no. / €) |
| Natural / chemical skin repellents (no. / €) |
| Mosquito traps (no. / €) |
| Larvicide (no. / €) |
| Mosquito nets (year of purchase / €) |
| Other products (no. / €) |
| CONDOMINIUM EXPENDITURES |
| YES / NO |
| Type of interventions |
| Expense (€) |
