# Supplementary material for: Household expenditure on control of urban mosquitoes Aedes albopictus and Culex pipiens in Emilia-Romagna, Northern Italy
Source: PLoS Negl Trop Dis. 2024 Oct 9;18(10):e0012552. doi: 10.1371/journal.pntd.0012552 (PMC11537423; doi:10.1371/journal.pntd.0012552)
Supplement: S2 Table — (DOCX) [file pntd.0012552.s003.docx]

**S2 Table. Stratified samples of candidate citizen interviewees by Local Health Authorities (LHAs) territory and municipality size and Distribution of interviews by LHAs territory and municipality size**

| Stratified samples of candidate citizen interviewees by Local Health Authorities (LHAs) territory and municipality size | | | | | | | | | |
| --- | --- | --- | --- | --- | --- | --- | --- | --- | --- |
| Population size of municipalities | Parma | Reggio E. | Bologna | Imola | Ravenna | Forlì | Cesena | Rimini | Total |
| <10,000 | 68 | 73 | 69 | 11 | 27 | 16 | 20 | 27 | 311 |
| 10,000-50,000 | 47 | 85 | 147 | 17 | 52 | 16 | 29 | 56 | 449 |
| >50,000 | 85 | 76 | 178 | 31 | 98 | 53 | 44 | 66 | 631 |
| Total | 200 | 234 | 394 | 59 | 177 | 85 | 93 | 149 | 1391 |
|  |  |  |  |  |  |  |  |  |  |
|  |  |  |  |  |  |  |  |  |  |
|  |  |  |  |  |  |  |  |  |  |
| Distribution of interviews by LHAs territorie and municipality size | | | | | | | | | |
|  |  |  |  |  |  |  |  |  |  |
| Number of interviews: |  |  |  |  |  |  |  |  |  |
| Population size | Parma | Reggio E. | Bologna | Imola | Ravenna | Forlì | Cesena | Rimini | Total |
| <10,000 | 16 | 21 | 24 | 6 | 7 | 5 | 7 | 7 | 93 |
| 10,000-50,000 | 13 | 30 | 41 | 5 | 17 | 6 | 10 | 14 | 136 |
| >50,000 | 22 | 22 | 48 | 11 | 32 | 16 | 12 | 20 | 183 |
| Total | 51 | 73 | 113 | 22 | 56 | 27 | 29 | 41 | 412 |
|  |  |  |  |  |  |  |  |  |  |
| % interviews |  |  |  |  |  |  |  |  |  |
| Population size | Parma | Reggio E. | Bologna | Imola | Ravenna | Forlì | Cesena | Rimini | Total |
| <10,000 | 3.9 | 5.1 | 5.8 | 1.5 | 1.7 | 1.2 | 1.7 | 1.7 | 22.6 |
| 10,000-50,000 | 3.2 | 7.3 | 10.0 | 1.2 | 4.1 | 1.5 | 2.4 | 3.4 | 33.0 |
| >50,000 | 5.3 | 5.3 | 11.7 | 2.7 | 7.8 | 3.9 | 2.9 | 4.9 | 44.4 |
| Total | 12.4 | 17.7 | 27.4 | 5.3 | 13.6 | 6.6 | 7.0 | 10.0 | 100.0 |
