# Supplementary material for: Household expenditure on control of urban mosquitoes Aedes albopictus and Culex pipiens in Emilia-Romagna, Northern Italy
Source: PLoS Negl Trop Dis. 2024 Oct 9;18(10):e0012552. doi: 10.1371/journal.pntd.0012552 (PMC11537423; doi:10.1371/journal.pntd.0012552)
Supplement: S3 Table — The table shows the results of the linear regression analysis of total spending for insect control, considering factors such as the level of annoyance related to different types of insects such as wasps, flies, common and tiger mosquitoes. Apart from columns (1) and (2) which analyze the full sample, the estimates in columns (3) and (4) pertain specifically to the subsamples of households with and without mosquito nets at home, respectively. In the full sample analysis in column (1), which excludes concern and bites annoyance from tiger mosquitoes, significant factors influencing total expenditure include the presence of an external garden, and nuisance levels from flies and common mosquitoes. In the full sample analysis in column (2), which includes all insects, significant factors positively affecting total expenditure are the presence of an external garden and the level of nuisance from flies. Additionally, households that experience higher levels of nuisance and concern from tiger mosquitoes tend to spend more on insect control measures. For households with mosquito nets (column 3), significant factors are the presence of an external garden, the area where the dwelling is located, the presence of flies, and the nuisance level from tiger mosquitoes. Higher education levels also significantly increase spending. In analysing the expenditure incurred by households without mosquito nets (column 4), significant factors include the presence of an external garden, the number of inhabitants, and the nuisance level from tiger mosquitoes. (DOCX) [file pntd.0012552.s004.docx]

**S3 Table. Linear regressions analysis of insect control expenditure (in log).**

|  | -1 | -2 | -3 | -4 |
| --- | --- | --- | --- | --- |
|  | Not including tiger mosquitoes | All insects | All insects with nets | All insects without nets |
| Floor number | -0.063 | -0.05 | -0.004 | -0.08 |
|  | [0.039] | [0.037] | [0.046] | [0.053] |
| External garden YN | 0.768^***^ | 0.628^***^ | 0.497^***^ | 0.918^***^ |
|  | [0.179] | [0.175] | [0.170] | [0.266] |
| Number of inhabitants | 0.074 | 0.086^*^ | -0.031 | 0.297^***^ |
|  | [0.054] | [0.051] | [0.044] | [0.100] |
| Presence of children | 0.168 | 0.002 | -0.005 | 0.364 |
|  | [0.152] | [0.149] | [0.164] | [0.291] |
| Urban centre | 0.224 | 0.032 | 0.901^***^ | -0.455 |
|  | [0.509] | [0.476] | [0.316] | [0.553] |
| Suburbs | -0.036 | -0.282 | 0.651^**^ | -0.855 |
|  | [0.506] | [0.476] | [0.317] | [0.571] |
| Countryside area | -0.033 | -0.175 | 0.625^**^ | 0.105 |
|  | [0.508] | [0.472] | [0.311] | [0.600] |
| Other locations (ref) |  |  |  |  |
|  |  |  |  |  |
| Flies nuisance lev. (ord) | 0.296^***^ | 0.207^**^ | 0.230^***^ | 0.112 |
|  | [0.099] | [0.089] | [0.085] | [0.196] |
| Wasps nuisance lev. (ord) | 0.156^*^ | 0.062 | 0.076 | 0.061 |
|  | [0.085] | [0.081] | [0.094] | [0.145] |
| Other insectc nuisance lev. (ord) | 0.102 | 0.113 | 0.231^**^ | -0.062 |
|  | [0.123] | [0.118] | [0.110] | [0.239] |
| Common mosq. nuisance lev. (ord) | 0.217^***^ | -0.055 | -0.047 | -0.151 |
|  | [0.076] | [0.081] | [0.072] | [0.216] |
| Tiger mosquito nuisance lev. (ord) |  | 0.438^***^ | 0.292^***^ | 0.535^***^ |
|  |  | [0.087] | [0.088] | [0.186] |
| Tiger mosquito concern lev. (ord) |  | 0.195^**^ | 0.115 | 0.299^*^ |
|  |  | [0.078] | [0.078] | [0.172] |
| Primary school (ref) |  |  |  |  |
|  |  |  |  |  |
| Lower secondary school | -0.025 | 0.047 | 0.016 | 0.239 |
|  | [0.195] | [0.187] | [0.148] | [0.392] |
| High school | -0.09 | -0.023 | 0.291^**^ | -0.401 |
|  | [0.192] | [0.181] | [0.145] | [0.374] |
| Bachelor degree | -0.22 | -0.076 | 0.491^**^ | -0.985^*^ |
|  | [0.464] | [0.414] | [0.235] | [0.585] |
| Master degree | -0.03 | -0.03 | 0.192 | -0.418 |
|  | [0.229] | [0.218] | [0.167] | [0.438] |
| Mosquito Nets YN | 0.874^***^ | 0.744^***^ |  |  |
|  | [0.128] | [0.120] |  |  |
| Constant | 2.232^***^ | 2.207^***^ | 2.496^***^ | 2.218^***^ |
|  | [0.556] | [0.517] | [0.353] | [0.673] |
| Observations | 295 | 294 | 170 | 124 |
| r2 | 0.381 | 0.44 | 0.356 | 0.412 |
| r2_a | 0.345 | 0.404 | 0.285 | 0.317 |
| bic | 917.082 | 896.587 | 409.696 | 460.188 |
| Robust standard errors in brackets |  |  |  |  |
| ^*^ *p* < 0.10, ^**^ *p* < 0.05, ^***^ *p* < 0.01 |  |  |  |  |
