# Supplementary material for: Household expenditure on control of urban mosquitoes Aedes albopictus and Culex pipiens in Emilia-Romagna, Northern Italy
Source: PLoS Negl Trop Dis. 2024 Oct 9;18(10):e0012552. doi: 10.1371/journal.pntd.0012552 (PMC11537423; doi:10.1371/journal.pntd.0012552)
Supplement: S4 Table — The table presents the results of the linear regression analysis of total expenditure on insect control, excluding costs related to the installation of mosquito nets. In the analysis on the full sample (column 1), significant factors influencing total expenditure include the presence of an external garden, the number of inhabitants, the nuisance level from mosquitoes, and the level of concern about their bites. For households with mosquito nets (column 2), significant factors are the nuisance level from insects and educational attainment. In households without mosquito nets (column 3), significant factors are the presence of an external garden, the number of inhabitants, the nuisance level from tiger mosquitoes, and the level of concern about their bites. These results indicate that the presence of an external garden and nuisance levels from insects consistently and positively impact insect control expenditure across all samples, with higher levels of concern also playing a significant role, particularly in households without mosquito nets. (DOCX) [file pntd.0012552.s005.docx]

S4 Table. Linear regression analysis of total expenditure on mosquito control (in log) net of mosquito net costs (OLS with robust s.e.)

|  | -1 | -2 | -3 |
| --- | --- | --- | --- |
|  | Full sample | With mosquito nets | Without mosquito nets |
| Floor number | -0.039 | -0.001 | -0.078 |
|  | [0.043] | [0.107] | [0.052] |
| External garden YN | 0.656^***^ | 0.554 | 0.925^***^ |
|  | [0.220] | [0.348] | [0.254] |
| Number of inhabitants | 0.109^*^ | 0.019 | 0.300^***^ |
|  | [0.064] | [0.082] | [0.099] |
| Presence of children | 0.155 | 0.107 | 0.389 |
|  | [0.232] | [0.290] | [0.290] |
| Urban centre | -0.202 | 0.199 | -0.452 |
|  | [0.439] | [0.379] | [0.525] |
| Suburbs | -0.484 | -0.001 | -0.864 |
|  | [0.436] | [0.331] | [0.544] |
| Countryside area | -0.388 | -0.024 | 0.143 |
|  | [0.464] | [0.336] | [0.577] |
| Other locations (ref) |  |  |  |
|  |  |  |  |
| Nuisance lev. (ord) | 0.512^***^ | 0.477^***^ | 0.459^***^ |
|  | [0.109] | [0.156] | [0.161] |
| Concern lev. (ord) | 0.278^**^ | 0.279 | 0.287^*^ |
|  | [0.133] | [0.187] | [0.169] |
| Primary school (ref) |  |  |  |
|  |  |  |  |
| Lower secondary school | 0.121 | 0.166 | 0.226 |
|  | [0.286] | [0.411] | [0.386] |
| High school | 0.28 | 0.800^**^ | -0.43 |
|  | [0.268] | [0.386] | [0.358] |
| Bachelor degree | 0.428 | 1.760^***^ | -0.951 |
|  | [0.495] | [0.418] | [0.611] |
| Master degree | 0.071 | 0.408 | -0.461 |
|  | [0.305] | [0.444] | [0.419] |
| Mosquito Nets YN | -0.286^*^ |  |  |
|  | [0.160] |  |  |
| Constant | 2.063^***^ | 1.395^**^ | 2.203^***^ |
|  | [0.531] | [0.631] | [0.656] |
| Observations | 294 | 170 | 124 |
| r2 | 0.211 | 0.192 | 0.408 |
| r2_a | 0.172 | 0.125 | 0.338 |
| Bic | 1069.432 | 649.251 | 441.625 |
| Robust standard errors in brackets |  |  |  |
| ^*^ *p* < 0.10, ^**^ *p* < 0.05, ^***^ *p* < 0.01 |  |  |  |
