# Supplementary material for: Household expenditure on control of urban mosquitoes Aedes albopictus and Culex pipiens in Emilia-Romagna, Northern Italy
Source: PLoS Negl Trop Dis. 2024 Oct 9;18(10):e0012552. doi: 10.1371/journal.pntd.0012552 (PMC11537423; doi:10.1371/journal.pntd.0012552)
Supplement: S5 Table — The estimation of the logit model reveals that the floor level negatively impacts this decision, suggesting that higher floors reduce the probability of purchasing nets. Conversely, living in a countryside area significantly increases the likelihood of investing in mosquito nets. Moreover, higher levels of nuisance from tiger mosquitoes and other insects positively influence the decision to install mosquito nets. Other factors, such as the presence of an external garden, the number of inhabitants, the presence of children, and concern level about tiger mosquitoes, do not appear to significantly affect the investment decision. (DOCX) [file pntd.0012552.s006.docx]

S5 Table. Logit model analysis of factors influencing investment in mosquito nets (binary dep. var.)

|  | Coeff. | s.e. |
| --- | --- | --- |
| Mosquito Nets YN |  |  |
| Floor number | -0.375^***^ | 0.108 |
| External garden YN | 0.239 | 0.334 |
| Number of inhabitants | 0.192 | 0.132 |
| Presence of children | 0.692 | 0.449 |
| Urban centre | 1.028 | 0.791 |
| Suburbs | 0.973 | 0.798 |
| Countryside area | 2.321^***^ | 0.851 |
| Other locations (ref) |  |  |
| Flies nuisance lev. (ord) | 0.09 | 0.263 |
| Wasps nuisance lev. (ord) | -0.265 | 0.286 |
| Other insects nuisance lev. (ord) | 0.568^*^ | 0.317 |
| Common Mosquitoes nuisance lev. (ord) | -0.376 | 0.241 |
| Tiger mosquito nuisance lev. (ord) | 0.641^***^ | 0.231 |
| Tiger mosquito concern lev. (ord) | 0.248 | 0.216 |
| Primary school (ref) |  |  |
| Lower secondary school | 0.409 | 0.489 |
| High school | -0.103 | 0.468 |
| Bachelor degree | -0.93 | 1.025 |
| Master degree | -0.295 | 0.517 |
| Constant | -1.676^*^ | 0.919 |
| Observations | 294 |  |
| r2 |  |  |
| Bic | 427.102 |  |
| Robust standard errors |  |  |
| ^*^ *p* < 0.10, ^**^ *p* < 0.05, ^***^ *p* < 0.01 |  |  |
